# A rare, spontaneous external jugular vein pseudoaneurysm: case report and literature review

**DOI:** 10.3389/fsurg.2026.1949023

**Published:** 2026-08-31

**Authors:** Yaohua Li, Ziqian Yin, Hongtao Zhang, Song Han

**Affiliations:** 1 Department of Neurosurgery, Tianjin Medical University General Hospital Tianjin, China,; 2 Tianjin Neurological Institute, Tianjin, China

**Keywords:** diagnosis, digital subtraction angiography, dsa, external jugular vein pseudoaneurysm, surgery

## Abstract

External jugular vein pseudoaneurysm represents an extremely rare venous lesion. We present the case of a 28-year-old female patient with a 2-year history of a left lateral neck mass that suddenly enlarged recently and was accompanied by severe pain. Digital subtraction angiography was applied to further confirm the venous origin of the lesion. The patient underwent complete surgical excision of the mass combined with ligation of the involved venous segment. This case indicates that external jugular vein pseudoaneurysm should be included in the differential diagnosis of lateral neck masses, particularly when the lesion presents with rapid enlargement and acute pain suggestive of secondary thrombosis. Multimodal imaging plays a crucial role in the accurate diagnosis of this rare disease, and complete surgical excision is a safe and effective therapeutic strategy for symptomatic lesions.

## Introduction

Due to hemodynamic factors, aneurysms and pseudoaneurysms predominantly occur in the arterial system, whereas true or false aneurysms arising from veins are exceedingly rare in clinical practice and are mostly iatrogenic in origin (1–3). Because of the low pressure within the venous system, venous aneurysms are most frequently encountered in the lower extremities (4). Pseudoaneurysms of the external jugular vein are even rarer than aneurysms, with farer fewer cases reported in the English-language literature.

Despite their low overall incidence, these lesions can present as rapidly enlarging masses over a short period, accompanied by high-risk complications such as rupture with hemorrhage, partial or complete thrombosis. Depending on the anatomical location, they may also give rise to significant cosmetic concerns. Clinically, patients typically present with neck swelling, pain, and tenderness. The accurate classification and optimal treatment strategies are often hampered by insufficient clinical awareness (5). Herein, we report a case of a spontaneous, rapidly enlarging pseudoaneurysm of the left external jugular vein in a 28-year-old woman, and review the relevant literature to discuss the diagnostic approach and treatment strategies for this rare condition.

## Case report

**Figure 1:**
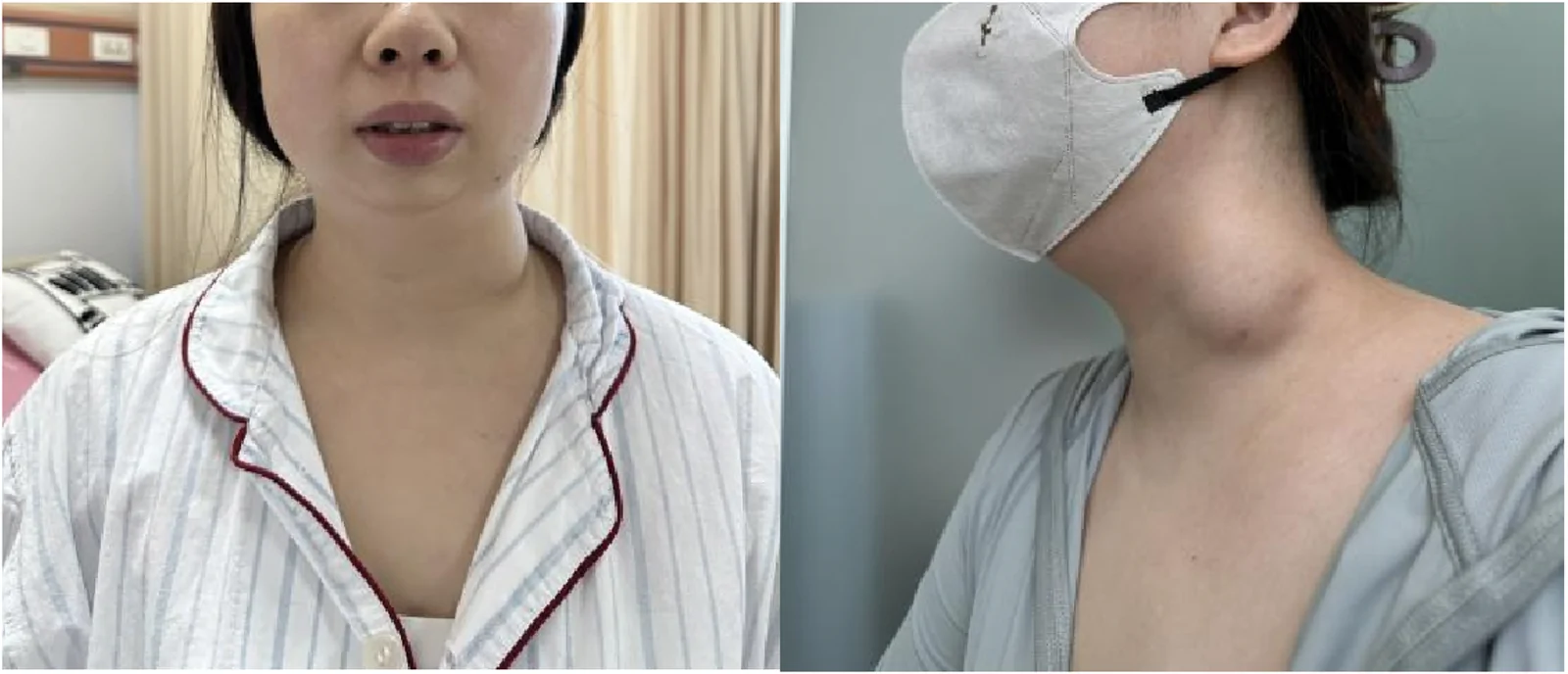
Pre-operative image of left neck mass.

A 28-year-old woman presented with sudden enlargement and pain of a subcutaneous mass in the left neck (Figure 1). The painless mass had been noted for 2 years; previous ultrasound had revealed a cystic lesion in the left neck concerning for an internal jugular vein aneurysm, and regular follow-up was advised. Two days before admission, the mass enlarged rapidly, accompanied by severe pain and numbness of the left neck and shoulder, which worsened with neck rotation. There was no dyspnea, and no history of neck trauma, venous catheterization, or neck surgery. Physical examination revealed a firm and tender neck mass without overlying skin pigmentation or inflammatory changes. On laboratory examination, the activated partial thromboplastin time was 33.6 s and the D-dimer level was 267 ng/mL, with no other hematologic abnormalities detected.

**Figure 2:**
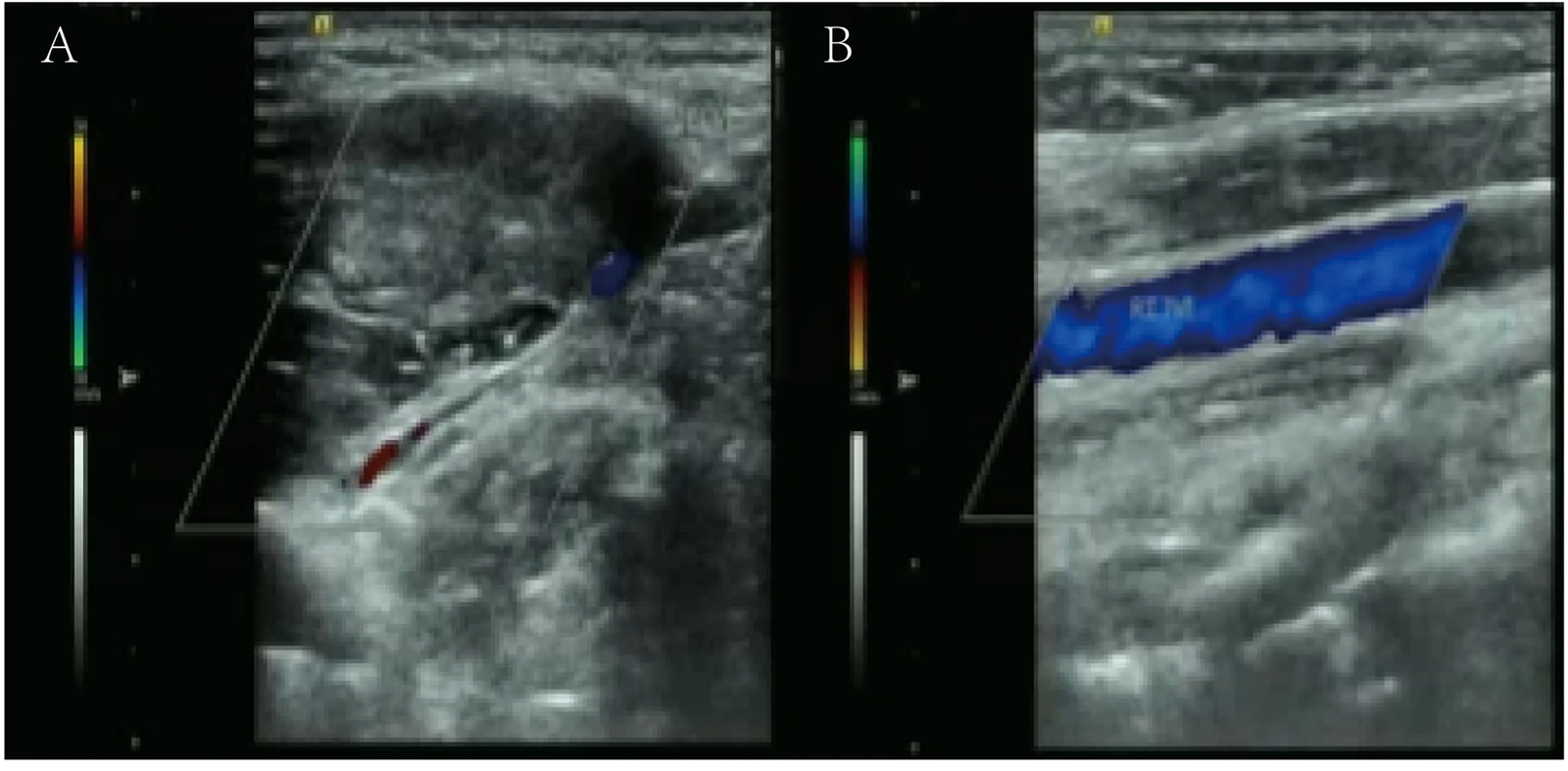
Pre-operative ultrasound imaging showing “cystic mass of the left neck”. **(A)** External jugular vein; **(B)** Internal jugular vein.

Ultrasonography demonstrated an ill-defined heterogeneous hypoechoic mass measuring approximately 4.6 * 4.2 * 1.8 cm in the left subcutaneous neck (Figure 2). The mass had well-defined borders and a regular shape, with no appreciable internal blood flow. It was closely related to the external jugular vein, causing local compression and flattening the vein. No flow communication between the mass and the external jugular vein was detected. The internal jugular vein was focally compressed and narrowed, but its wall was not thickened, and no intraluminal thrombus was observed; flow within the internal jugular vein remained relatively patent.

**Figure 3:**
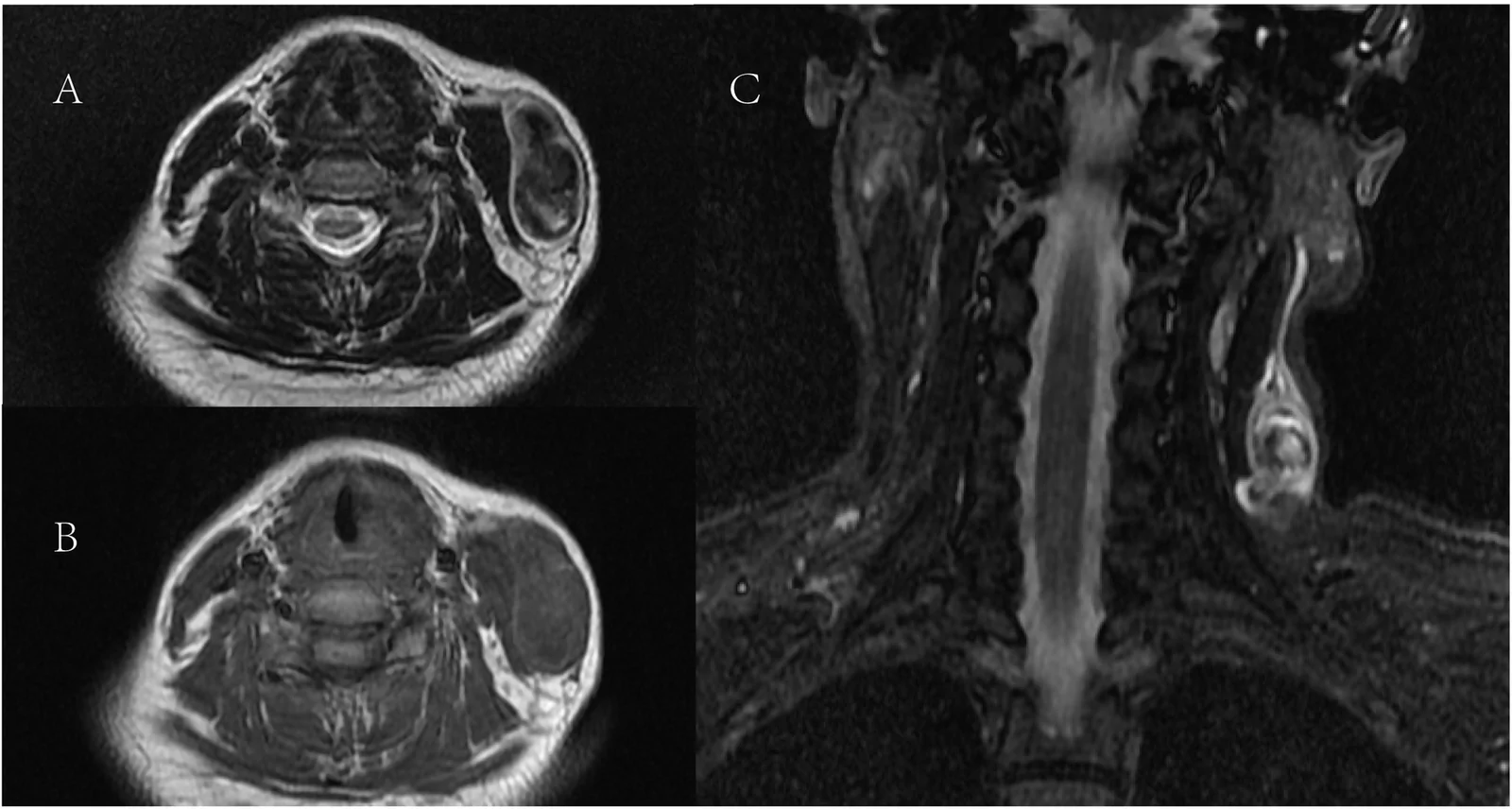
Pre-operative the MRI scan findings of the neck soft tissue. A: Axial T2; B: Axial T1; C: Coronal T2 STIR.

Magnetic resonance imaging of the neck revealed a well-circumscribed mass in the subcutaneous soft tissue lateral to the left sternocleidomastoid muscle (Figure 3). On T1-weighted imaging, the lesion was predominantly isointense to the muscle with patchy slightly hyperintense areas. T2-weighted imaging showed a heterogeneous signal pattern, characterized by a predominantly hypointense background interspersed with foci of variable signal intensity. The lesion exerted a significant mass effect, compressing and medially displacing the adjacent left sternocleidomastoid muscle. The fat-suppressed sequence revealed a fusiform cystic lesion with hyperintense fluid signal in the left supraclavicular region. The upper airway was unremarkable, without epiglottic thickening; the pyriform sinuses were symmetrical; the aryepiglottic folds showed normal morphology and signal intensity; the vocal cords were symmetrical; and the tracheal lumen was patent without evidence of significant compression or displacement. Multiple small lymph nodes measuring less than 1 cm in diameter were noted in the bilateral submandibular, jugulodigastric, posterior cervical, supraclavicular, and parotid regions. Cerebral angiography demonstrated a ball-holding sign at the junction of the left internal jugular vein and external jugular vein (Supplementary Video S1).

**Figure 4:**
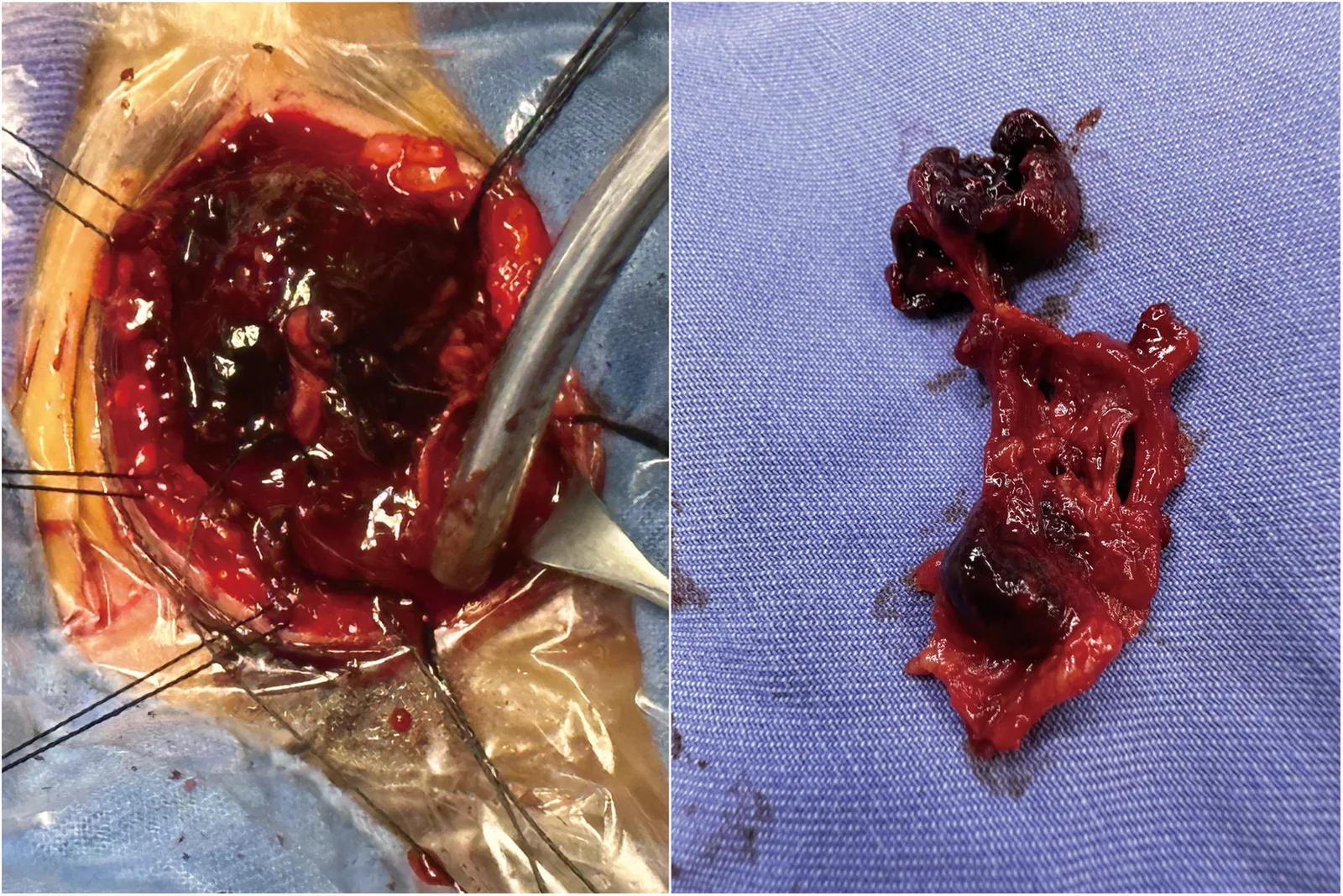
The intraoperative observations during surgical exploration.

**Figure 5:**
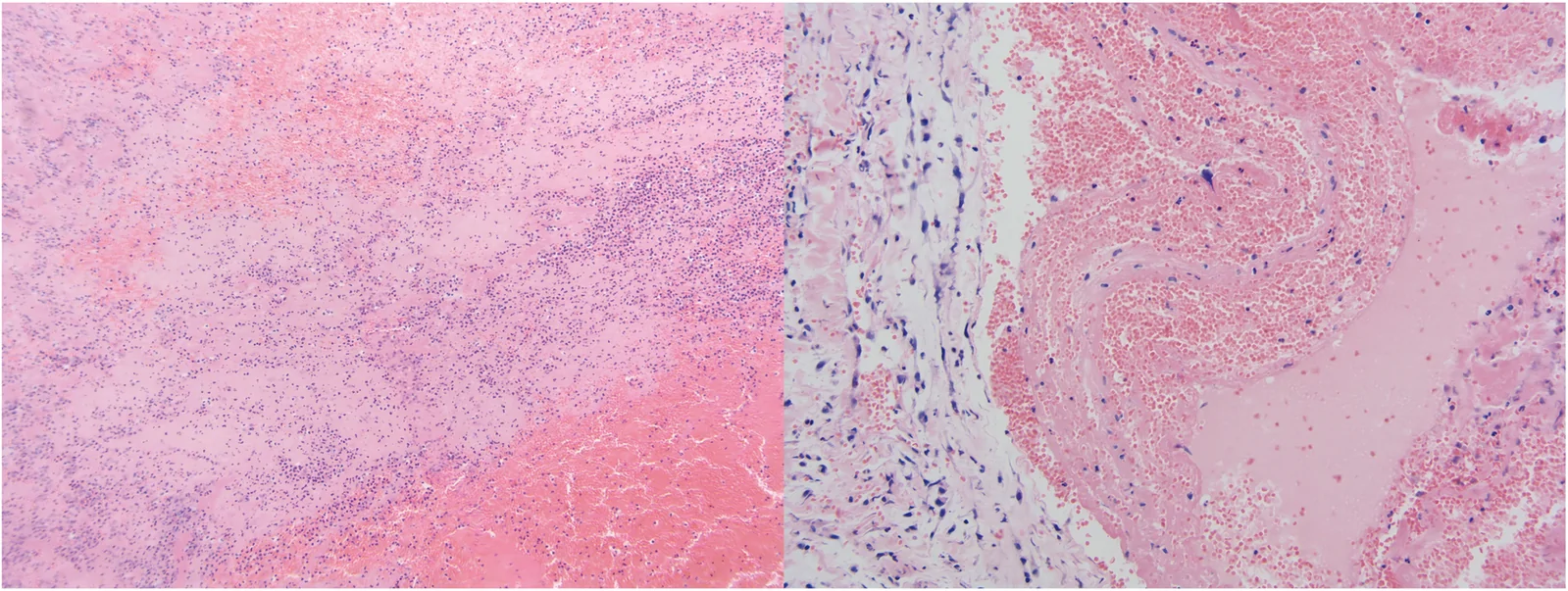
The histopathological analysis from the post-operative specimen using Hematoxylin and Eosin (H&E) staining.

Surgical excision of the external jugular vein pseudoaneurysm was performed under general anesthesia. Intraoperative exploration revealed a mass located superficial to the left sternocleidomastoid muscle (Figure 4). The lesion was encapsulated, with intact and well-defined vessel wall structures at its periphery. A hematoma was observed beneath the capsule, showing evidence of partial organization and active venous bleeding. The aneurysm was carefully ligated proximally and distally, and then excised completely. The hematoma and organized tissue in the neck were evacuated, and the wound was closed meticulously in layers. The patient recovered uneventfully and was discharged three days postoperatively. During 3-month follow-up, the neck swelling was significantly reduced, and the neck pain and numbness resolved completely with restoration of a full range of neck motion. Pathological examination suggested venous thrombosis accompanied by early organization (Figure 5).

**Figure 6:**
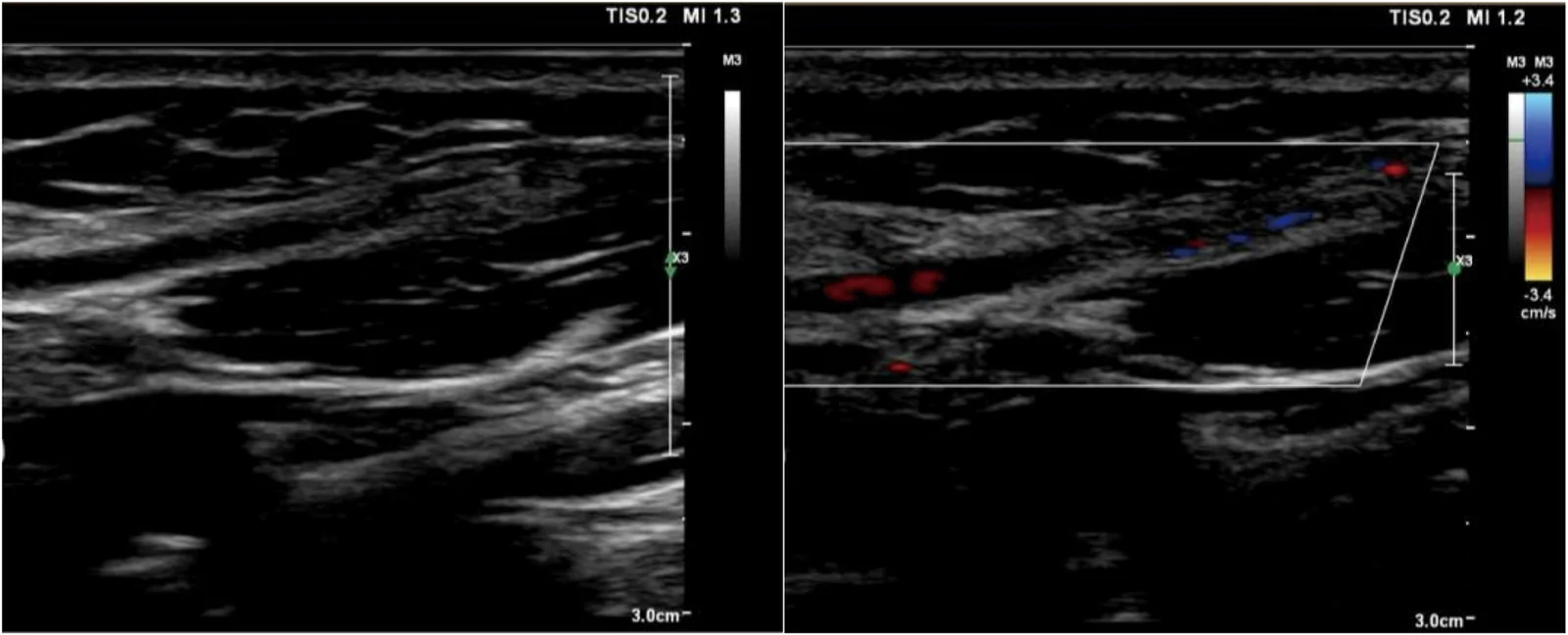
Post-operative ultrasound examination.

Postoperative Doppler ultrasonography confirmed the absence of the left external jugular vein at the surgical site, consistent with surgical ligation and resection. The thrombus completely occluded the residual venous segment, and color Doppler imaging confirmed the absence of flow, indicating complete thrombosis and functional occlusion of the proximal venous segment (Figure 6).

## Discussion

The causes of venous aneurysms can be classified as primary and secondary (6). Venous aneurysms are often secondary to various factors that alter venous system pressure or flow velocity, including trauma, external compression by tumors or cysts, infection, inflammation, and iatrogenic or behavioral injuries such as central venous catheter placement and intravenous drug abuse (1–3). Compared with the veins of the lower extremities, the cervical veins are located in a region of lower pressure, have more distensible walls, and lack venous valves; consequently, the incidence of thrombosis and aneurysm formation at this site is relatively low (7). A true aneurysm is pathologically characterized by aneurysmal dilatation involving all three layers of the vessel wall; a pseudoaneurysm, in contrast, results from a breach in the vessel wall with extravasation of blood that is contained by surrounding tissues, and it does not contain intact vascular wall layers (4). We report an extremely rare case of spontaneous venous pseudoaneurysm of the external jugular vein in a young woman, which was successfully treated surgically.

A PubMed search using the keywords “spontaneous”, “pseudoaneurysm”, “external jugular vein”, and “English” identified six relevant articles. In the reports by Parikh and Chapman, postoperative pathological examination supported the diagnosis of venous aneurysm. Among the remaining four cases, only one underwent open surgical treatment. Notably, all reported patients were female. Details are summarized in Table 1 (1, 3, 8, 9). To the best of our knowledge, this is the only reported case that underwent preoperative DSA evaluation and was successfully managed by surgical resection.

**Table 1:** Literature review of external jugular vein pseudoaneurysm.

Author	Year	Gender/Age	Size (cm)	Past medical history	Treatment
Ioana Grigorescu	2012	F/58	3.5*3.2*3	Primary arterial hypertension, chronic atrial fibrillation, mitral and aortic regurgitation, and moderate pulmonary hypertension	Conservative
Patrick J. Wallace	2019	F/27	1.7*1.4*2	–	Conservative
Dino Papes	2023	F/2	4*2.5	–	Surgery
Sebahat N. Dogan	2019	F/57	2.4*0.9	–	Percutaneous thrombin injection

Because involvement of the external jugular vein is exceedingly rare, standardized diagnostic and therapeutic protocols remain lacking. Imaging plays a critical role in the differential diagnosis. Color Doppler ultrasonography is non-invasive, convenient, and cost-effective, and should serve as the screening modality of choice; CT angiography or magnetic resonance imaging can further delineate the anatomical relationship between the lesion and the parent vein, the morphology of the aneurysm, and the presence or absence of mural thrombus. Although DSA is an invasive examination and is not routinely used as the first-line modality, in the present case it played an important role in further defining the hemodynamic characteristics of the lesion, clarifying its relationship with the internal and external jugular veins, excluding other vascular lesions, and informing the subsequent treatment strategy. For neck masses without a definite history of trauma, the differential diagnosis should include lymphadenopathy, cystic thyroid nodules, branchial cleft cysts, thyroglossal duct cysts, and the rare venous aneurysms. Based on the available literature and our clinical experience, for asymptomatic head and neck pseudoaneurysms in patients who decline or are unsuitable for invasive intervention, conservative management or thrombin injection of urokinase may be considered. However, when the lesion exhibits progressive enlargement in conjunction with severe pain, this may be indicative of rebleeding or wall rupture, and early open surgical treatment should be considered.

Complications of venous aneurysms, though exceedingly rare, include thromboembolism, impaired venous drainage, rupture, and compression of adjacent structures, and can be life-threatening once they occur. When a mass develops pain, tenderness, or rapid enlargement over a short period, intra-aneurysmal thrombosis or impending rupture should be highly suspected (4); in our patient, both intraoperative findings and imaging confirmed the presence of thrombus. Available evidence indicates that anticoagulation therapy alone is insufficient to prevent the above-mentioned complications (10–13). Therefore, treatment should be stratified according to symptoms and the rate of progression: for asymptomatic, stable lesions, periodic follow-up observation is a reasonable option; whereas for lesions that are rapidly enlarging, accompanied by pain or thrombosis, or causing cosmetic concerns, complete surgical excision with ligation of the involved venous segment has become the generally accepted treatment approach (14–20).

Overall, the diagnosis and management of external jugular vein pseudoaneurysm require close integration of clinical presentation and imaging features. Given the limited published literature, no precise guidelines are currently available for specific cases, and treatment decisions should be individualized based on a careful balance between complication risk and interventional benefit, while respecting patient preference.

## Conclusion

We report the first case of spontaneous pseudoaneurysm of the external jugular vein with comprehensive DSA evaluation. This case highlights that such lesions should be considered in lateral neck masses, particularly when acute pain or rapid enlargement suggests thrombosis. Complete surgical excision with ligation is safe and effective, and management should be individualized based on clinical and imaging findings.

## Data Availability

The original contributions presented in the study are included in the article/Supplementary Material, further inquiries can be directed to the corresponding author/s.
